# Improving the Hip Fracture Risk Prediction Through 2D Finite Element Models From DXA Images: Validation Against 3D Models

**DOI:** 10.3389/fbioe.2019.00220

**Published:** 2019-09-10

**Authors:** Mara Terzini, Alessandra Aldieri, Luca Rinaudo, Giangiacomo Osella, Alberto L. Audenino, Cristina Bignardi

**Affiliations:** ^1^Polito^BIO^Med Lab, Department of Mechanical and Aerospace Engineering, Politecnico di Torino, Turin, Italy; ^2^TECHNOLOGIC S.r.l. Hologic Italia, Turin, Italy; ^3^Department of Clinical and Biological Sciences, Internal Medicine, San Luigi Gonzaga University Hospital, Orbassano, Italy

**Keywords:** osteoporosis, hip fracture, fracture risk, finite element analysis, DXA, CT

## Abstract

Osteoporotic fracture incidence represents a major social and economic concern in the modern society, where the progressive graying of the population involves an highly increased fracture occurrence. Although the gold standard to diagnose osteoporosis is represented by the T-score measurement, estimated from the Bone Mineral Density (BMD) using Dual-energy X-ray Absorptiometry (DXA), the identification of the subjects at high risk of fracture still remains an issue. From this perspective, the purpose of this work is to investigate the role that DXA-based two-dimensional patient-specific finite element (FE) models of the proximal femur, in combination with T-score, could play in enhancing the risk of fracture estimation. With this aim, 2D FE models were built from DXA images of the 28 post-menopausal female subjects involved. A sideways fall condition was reproduced and a Risk of Fracture ($\hat{\text{RF}}$) was computed on the basis of principal strains criteria. The identified $\hat{\text{RF}}$ was then compared to that derived from the CT-based models developed in a previous study. The 2D and 3D $\hat{\text{RF}}$ turned out to be significantly correlated (Spearman's ρ = 0.66, *p* < 0.001), highlighting the same patients as those at higher risk. Moreover, the 2D $\hat{\text{RF}}$ resulted significantly correlated with the T-score (Spearman's ρ = −0.69, *p* < 0.001), and managed to better differentiate osteopenic patients, drawing the attention to some of them. The Hip Structural Analysis (HSA) variables explaining the majority of the variance of the 2D and 3D fracture risk were the same as well, i.e., neck-shaft angle and narrow neck buckling ratio. In conclusion, DXA-based FE models, developable from currently available clinical data, appear promising in supporting and integrating the present diagnostic procedure.

## Introduction

During the last decades osteoporotic fractures have grown into a major healthcare concern, especially in western countries. They are increasingly becoming a considerable cause of mortality and morbidity (Cooper et al., 1993; Dennison et al., 2005), exacerbated by the growing number of elderly individuals (Sambrook and Cooper, 2006). It is estimated that, in Europe, 30% of women over 50 years are affected by osteoporosis (Melton et al., 1992; Kanis et al., 2000; Hernlund et al., 2013) and that in 2000 the number of osteoporotic fractures was 3.1–3.7 million, with a direct cost of 32 billion (Kanis and Johnell, 2005). In addition, these costs are expected to raise to more than double by 2050 (Kanis and Johnell, 2005). The hip is recognized as the most serious fracture, with a 20% mortality rate in the first year following the fracture and 50% of the patients suffering from a reduced functional capacity (Cooper et al., 1993). Although loss of mechanical strength associated to the onset of osteoporosis predisposes the proximal femur to fracture, it is still not trivial to interpret the physiopathology of osteoporotic fractures, which represent complex multifactorial events.

At present, the gold standard for osteoporosis diagnosis is represented by the Dual X-ray Absorptiometry (DXA) imaging technique. Projecting the three-dimensional bone structure on a plane, DXA yields a two-dimensional image (D'Amelio et al., 2008) from which the BMD measurement is extracted. Comparing the subject-specific BMD value with the average value of a young standard population in terms of standard deviations, the T-score is derived. The T-score represents the clinical parameter on which the discrimination between pathological and physiological individuals is based. Nevertheless, it does not account for crucial structural parameters such as the bone geometry and internal architecture (Mccreadie and Goldstein, 2000) and it has been shown to suffer from a limited sensitivity in the prediction of fracture (Kanis et al., 2011). Pharmacological treatments, especially those acting on cortical thickness (Häuselmann and Rizzoli, 2003; Watkins et al., 2012), have demonstrated to reduce the risk of fracture (Kanis et al., 2019); therefore, the accurate identification of subject at higher risk represents a pivotal issue. From this perspective, many authors have extensively focused on the fracture prediction enhancement. On one hand, efforts have been put in the identification of geometric features (Beck, 2007; Beck and Broy, 2015) predisposing proximal femur to fracture (Cooper et al., 1993; Gregory and Aspden, 2008; Kaptoge et al., 2008; Ito et al., 2010; Luo et al., 2011; Gnudi et al., 2012; Danielson et al., 2013; Aldieri et al., 2018). Commonly, this has been carried out considering Hip Structural Analysis (HSA) parameters (Beck, 2007), geometric variables measured on DXA images which approximately describe the patient-specific geometry. On the other hand, the use of Finite Element (FE) analysis has been proposed as a powerful and reliable computational tool able to comprehensively estimate fracture risk (Schileo et al., 2008b; Luo et al., 2011, 2018; Op Den Buijs and Dragomir-Daescu, 2011; Koivumäki et al., 2012; Naylor et al., 2013; Yang et al., 2014, 2018; Dall'Ara et al., 2016; Grassi et al., 2016; Bhattacharya et al., 2018). Three-dimensional models derived from CT scans have been shown to provide accurate results compared to experimental tests, being able to correctly predict fracture onset (Schileo et al., 2008b; Koivumäki et al., 2012; Bhattacharya et al., 2018). CT-based models manage indeed to closely reflect not only the 3D subject-specific geometry of the proximal femur, but also the three-dimensional distribution of its inhomogeneous mechanical properties. Nevertheless, because of the associated X-rays dose and costs, CT does not represent the first choice exam for osteoporosis screening purposes. This is the reason why the role of 2D FE models developed starting from DXA images has also been investigated (Luo et al., 2011, 2018; Op Den Buijs and Dragomir-Daescu, 2011; MacNeil et al., 2012; Naylor et al., 2013; Yang et al., 2014, 2018; Dall'Ara et al., 2016). In spite of DXA projective nature, which provides a two-dimensional simplified representation of a complex three-dimensional structure, DXA images are indeed routinely acquired and thus clinically available. FE model outcomes have been shown to potentially enhance fracture risk estimation, bringing additional information independently from BMD (Taylor et al., 2004; Yang et al., 2014, 2018; Dall'Ara et al., 2016; Luo et al., 2018). Therefore, aiming to get further insights in the role 2D FE simulations might play, the main purpose of the study was the comparison between the predictions, in terms of risk of fracture, derived from the CT (Aldieri et al., 2018) and DXA FE models. This could be achieved thanks to the simultaneous availability of CT and DXA images for the same patients, which rarely occurs. Furthermore, the consistency between 2D and 3D FE analyses outcomes was investigated also from the perspective of the HSA variables. They represent indeed available geometric descriptors potentially able to integrate the T-score which carries information regarding only the average material properties.

## Materials and Methods

A cohort composed of 28 post-menopausal female subjects, aged from 55 to 81 years (treated in San Luigi Gonzaga Hospital in Orbassano, Italy), was included in the present study after having signed an informed consent. Since the possible presence of bone metastasis might have affected bone strength, patients affected by cancer were not involved. Thanks to cross checks within the Hospital Database, clinical and DXA-derived data (acquired with a Discovery DXA system, Hologic), together with CT scans, were available for the whole cohort. Therefore, 2D FE models were set up starting from DXA images, with the purpose of comparing their predictive capabilities with those of 3D models built from CT data in a previous work (Aldieri et al., 2018). Follow-up information for these 28 patients was not available, so, it was not possible to know if they actually ever fractured. For this reason, two additional post-menopausal female patients aged 71 and 75 years, fractured at the proximal femur within 1 year after the DXA exam, were included in the cohort, although the related 3D models construction was prevented by the lack of the CT scans.

### Local BMD Map Definition

DXA scans of the proximal femur were first segmented through a semi-automatic procedure. Subsequently, because the material properties definition is BMD-dependent, a procedure for assigning patient-specific local BMD values was developed, postulating a linear relationship between pixels gray levels and BMD. The pixel-by-pixel BMD map was indeed not available and Hologic software provided average BMD values restricted to only three Regions of Interest (ROI), i.e., neck, trochanter and intertrochanter (Figure S1). Specifically, accounting for each ROI individually, the unknown minimum BMD value, equivalent to the line intercept, was determined in five different trials as 10, 15, 20, 25, and 30% of the average BMD. Non-zero pixel gray values were then averaged to find the gray value corresponding to the ROI-specific mean BMD measurement provided by DXA, in order that the line slope could be determined as well. Aiming at the definition of a unique patient-specific linear relation between gray values and BMD, the intercepts and slopes obtained working on each ROI individually were eventually averaged. Further details about the BMD mapping procedure from the DXA image gray levels are provided in the Supplementary Material.

### FE Analyses

Two-dimensional FE analyses were set up assuming the proximal femur was a plate with a constant thickness. Plane stress elements were used, considered to be the most adequate with respect to both femur three-dimensional structure and sideways loading conditions (Naylor et al., 2013; Luo et al., 2018). After performing a sensitivity analysis taking into account the highest values of tensile and compressive principal strains, element size was set to 0.5 mm. The plate thickness was assigned in a patient-specific way, according to Naylor et al. (2013), and computed from the femoral neck width included in Hip Structural Analysis (HSA) data. Specifically, it was determined such that its cross section area and moment of inertia matched those of a circular cross section at the femoral neck. Subsequently, using the patient-specific plate thickness and the previously defined BMD local map, local material properties could be defined. First of all, the areal BMD values were converted to volumetric density values using the previously defined patient-specific plate thickness (Martin and Burr, 1984; Naylor et al., 2013); afterwards, volumetric density was converted to apparent density (ρ_*app*_) according to Schileo et al. (2008b). Eventually, Young's moduli (*E*) were derived using the empirical relations developed by Morgan et al. (2003):

$$E\left(MPa\right)=\left\{\begin{array}{c} 15010\rho_{app}{}^{2.18}\;if\;\rho_{app}\leq 0.28\;g/cm^{3}\\ 6850\rho_{app}{}^{1.49}\;if\;\rho_{app}>0.28\;g/cm^{3} \end{array}\right\}$$

The Young's moduli range was further divided in 35 bins in which mesh elements were grouped according to their corresponding closest pixel gray value; a Young's modulus equivalent to the median value of the bin they belonged to was then assigned to each element.

**Figure 1:**
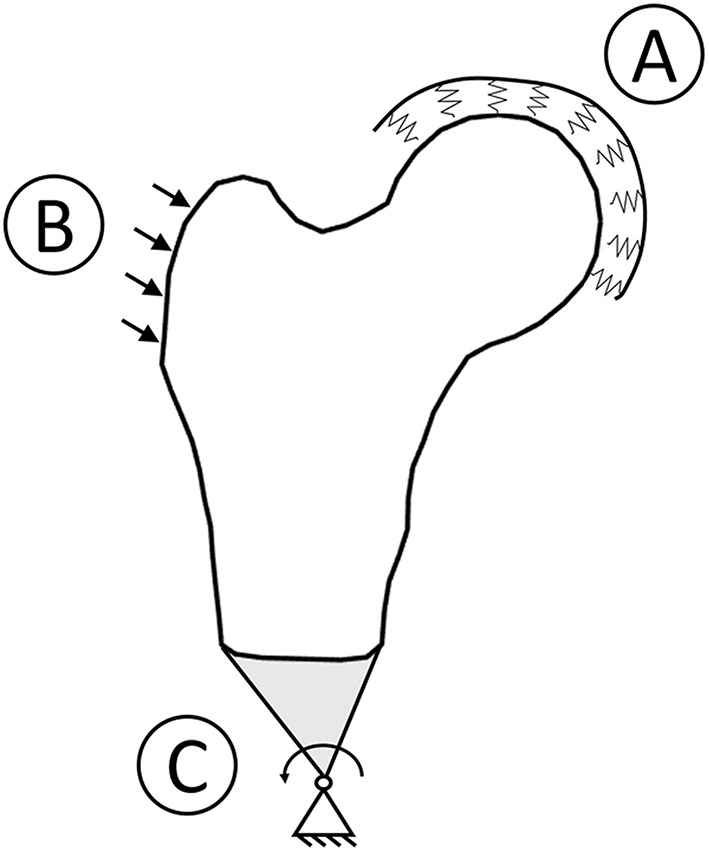
Boundary conditions graphic overview. To simulate a sideways fall condition, the head was bound to the ground using spring element **(A)**, the impact force was applied with a 30° angle with respect to the shaft perpendicular axis **(B)**, and the distal femur was connected to a hinge located distally **(C)**.

Boundary conditions reproducing a sideways fall condition were applied coherently with the previous study (Aldieri et al., 2018). Briefly, prior to the FE analysis, a 1 Degree of Freedom spring mass damper system was solved accounting for subject-specific height and weight, aiming to estimate subject-specific impact force to be applied on trochanteric surface. Then, the FE simulations were set up: the impact force was applied on the frontal plane, inclined 30° counter clockwise with respect to the shaft perpendicular axis. The femoral head was bound to the ground by means of spring elements with a $10000\;\frac{N}{mm}$ stiffness, while distal nodes were connected to a node located distally, with rotational degrees of freedom only (Figure 1). Principal strains based criteria (Schileo et al., 2008b, 2014; Grassi et al., 2012) were adopted to predict fracture. In particular, at each element centroid, a Risk of Fracture (RF) was computed dividing the prevailing principal strain, evaluated comparing tensile and compressive principal strains, by the respective threshold value (Bayraktar et al., 2004). For each patient, the maximum RF value, addressed in the following as $\hat{\text{RF}}$, was identified excluding head and trochanteric regions (Schileo et al., 2014; Bhattacharya et al., 2018), where results could have been affected by boundary conditions. $\hat{\text{RF}}$ values exceeding the 99.9th percentile computed on the RF of the whole cohort were recognized as fracture prognostic. Differently from the 3D case, where only cortex elements were considered when identifying the $\hat{\text{RF}}$ (Aldieri et al., 2018), the whole elements set was here taken into account due to the projective nature of the DXA images and thus of the models.

For sake of comparison with the 3D models outcomes, a preliminary comparison between 2D and 3D models was carried out taking into account the averaged material properties and the computed $\hat{\text{RF}}$. On one hand, it was done aiming to assess if the initial hypothesis of a linear relation between gray levels and BMD was legitimate; on the other hand, to assess which estimate of the minimum BMD value among those adopted (10, 15, 20, 25, and 30% of the average BMD) best matched 3D results. In addition, geometric HSA parameters were also taken into account. Because these measurements are currently available in clinics and, as already mentioned, geometry contributes to overall femur strength, HSA parameters most meaningful to the CT-based $\hat{\text{RF}}$ were computed in the previous work (Aldieri et al., 2018). Therefore, for comparison purposes, HSA variables most relevant to the 2D $\hat{\text{RF}}$ were here identified as well. First of all, a collinearity diagnosis based on Variance Inflation Factor (VIF) (Walker, 1989) calculation was carried out, so that only independent HSA parameters were selected. Subsequently, Akaike Information Criterion (AIC) (Akaike, 1974) was adopted to identify those most related to the 2D $\hat{\text{RF}}$. Further details about HSA and AIC can be found in Aldieri et al. (2018).

## Results

Patient-specific Young's moduli averaged on the whole 2D models turned out to be all significantly correlated (*p* < 0.05) with 3D derived average elastic moduli, independently from the adopted estimate of the minimum BMD value. As far as the $\hat{\text{RF}}$ values are concerned, on which the predictive power for both the 2D and 3D models was based, the significant correlation (*p* < 0.01) between them was preserved regardless the chosen definition of the minimum BMD value. The obtained minimum BMD values were also compared to values reported in literature (Keller, 1994; Keyak et al., 1994; Ruess et al., 2012): values corresponding to 30% of the mean BMD resulted to be too high and the linear relations built on those were thus neglected in the subsequent analyses. The remaining linear relations provided minimum BMD values in accordance with the literature as well as analogous results also in terms of the $\hat{\text{RF}}$ trends and the most relevant HSA variables. The linear relation assuming the least BMD value equivalent to 20% of the average one led, besides, to the highest and most significant correlation with respect to the 3D $\hat{\text{RF}}$ (Spearman's ρ = 0.66, *p* < 0.001). Therefore, the corresponding local material properties definition was judged the optimal one, and only the outcomes associated to the aforementioned material properties definition will be presented in the following.

**Figure 2:**
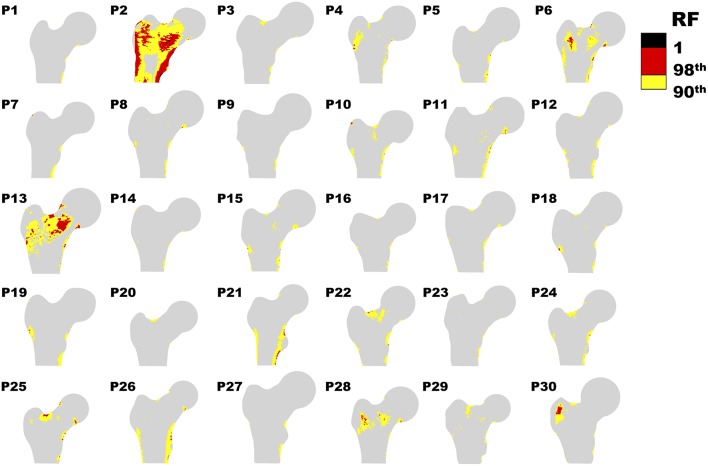
Contour plot representing RF distribution for each patient. Only RF above the 90th percentile (0.23) are shown.

**Figure 3:**
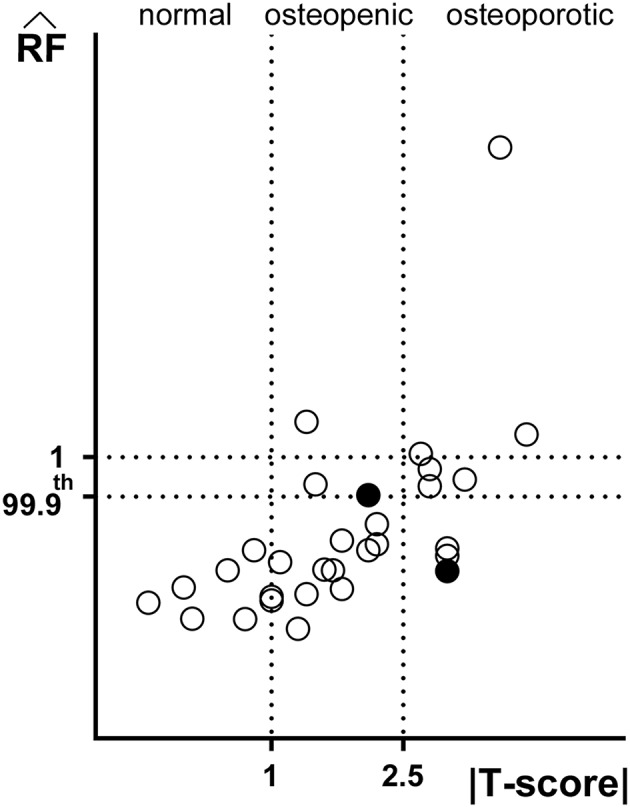
Comparison between predictive outcomes of |T-score| and $\hat{\text{RF}}$ (Spearman's ρ = 0.69, *p* < 0.001). The standard ranges of the T-score based criterion |T-score| <1 normal, 1 < |T-score| <2.5. osteopenic and |T-score| > 2.5 osteoporotic are highlighted. Filled circles refer to the two fractured patients' $\hat{\text{RF}}$. Not only $\hat{\text{RF}}$ values exceeding 1, but also those exceeding the 99.9th percentile have been regarded as fracture prognostic.

**Figure 4:**
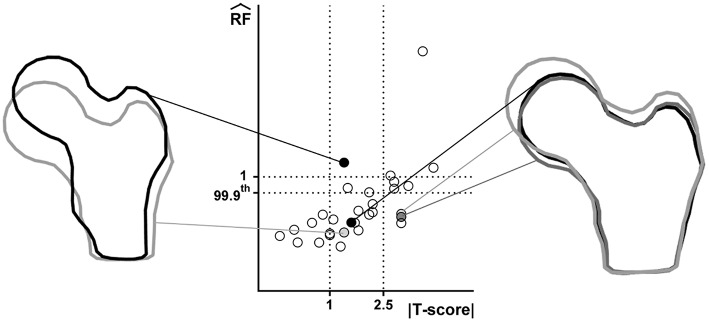
Juxtaposition of shapes related to patients with comparable T-score but different $\hat{\text{RF}}$ and vice versa. Considered patients are highlighted in the T-score-$\hat{\text{RF}}$ graph through filled circles. Right: three patients with similar $\hat{\text{RF}}$ but different T-score; left: two osteopenic patient with different $\hat{\text{RF}}$.

Figure 2 offers a graphic overview of the whole dataset, displaying contour maps of the RF exceeding the 90th percentile of the entire RF values set (corresponding to a RF = 0.23). The choice of including elements with RF values between the 98th percentile (RF = 0.45) and 1 in the medium risk range was made for sake of visualization only. If compared with the corresponding outcomes resulting from the three-dimensional analyses (Aldieri et al., 2018), the patients highlighted owing to a significant number of elements with RF exceeding the 90th percentile do not differ considerably (Figure S3). Patients 2, 6, 13 and, even though more slightly, 25, manage indeed to qualitatively stand out from the others in both 2D and 3D models. Beyond this visual comparison, it must be pointed out that the risk of fracture quantitative assessment was performed accounting for the maximum RF value (i.e., the $\hat{\text{RF}}$). Hence, there might be patients with a limited number of elements with RF exceeding the 90th percentile although exhibiting relatively high $\hat{\text{RF}}$ pertaining to a restricted region. From this perspective, Figure 3 shows the comparison between the risk of fracture as predicted through the gold standard T-score and through the $\hat{\text{RF}}$ coming from the 2D FE analyses. As visible, patients are gathered in a pretty narrow $\hat{\text{RF}}$ range with respect to T-score patient-specific data. As far as the risk assessment is concerned, patients characterized by $\hat{\text{RF}}$ exceeding the 99.9th percentile of the whole RF dataset, corresponding to 0.87, were considered to be at risk of fracture. Individuals with a non-pathological T-score all resulted in low $\hat{\text{RF}}$ values, while among osteopenic individuals, although some did not differentiate much from healthy ones in terms of the $\hat{\text{RF}}$, some others appeared at higher risk of proximal femur fracture. Eventually, although the majority of osteoporotic individuals were found within the high $\hat{\text{RF}}$ region, three of them, including one of the two fractured patients, did not result at risk based on the $\hat{\text{RF}}$. The reason clarifying why the aforementioned fractured patient was not classified as at risk by the $\hat{\text{RF}}$ might lie in her extremely low BMI (Table S1), on which the impact force applied in the FE simulations was dependent. Besides, further explanations can be achieved examining patient-specific proximal femurs geometries, which certainly play a role within FE analysis. In Figure 4, shapes of patients characterized by a similar T-score but a different $\hat{\text{RF}}$, or vice versa, are compared. Considering the profiles of the two osteoporotic patients not identified as at risk by the $\hat{\text{RF}}$ with respect to an additional osteopenic patient located in the same low $\hat{\text{RF}}$ region, they do not display significantly visible geometric differences. Similarly, examining the profiles of two patients with similar T-score (osteopenic range) but very different $\hat{\text{RF}}$ values, their profiles noticeably differ much more. This certainly directs attention to the synergic effect geometry and bone mineral density had in determining overall bone strength within FE analyses. Among the individuals identified as at high risk of fracture, which were 9, the fracture initiation could be speculated according to the location of the $\hat{\text{RF}}$, which was extracapsular for 5 individuals, while being intracapsular for the other 4 patients.

Aiming to identify the most significant HSA parameters with respect to the $\hat{\text{RF}}$, those explaining the majority of its variance were selected adopting the AIC (Akaike, 1974), after diagnosing collinearity within the whole set through calculation of the VIF (Walker, 1989). VIF calculation allowed the selection of 11 variables out of the 20 original ones. Interestingly, the most meaningful geometric parameters were represented by the Neck Shaft Angle (NSA), Buckling Ratio (BR) and Width computed at the narrow neck (*p* < 0.05), which were in agreement with the outcomes of the 3D models. The ranking of the non-collinear variables, carried out computing their cumulative Akaike weights which act as surrogates of their relative relevance (Gallo et al., 2012; Aldieri et al., 2018), is presented in Table 1. Table S1 also presents patient-specific values of the 11 non-collinear variables.

**Table 1:** The 11 non collinear HSA variables ranked according to their corresponding cumulative Akaike weights: Neck Shaf Angle (NSA), Buckling Ratio (BR), Width (W), Cross-sectional Moment of Inertia (CSMI), Hip Axis Length (HAL).

**HSA variable**	**Cumulative weight**
NSA	0.987
NN BR	0.983
NN W	0.470
NN CSMI	0.332
HAL	0.303
FS CSMI	0.244
IT W	0.212
IT BR	0.202
IT CSMI	0.194
FS W	0.190
FS BR	0.184

*NN, IT, FS refer to the three locations where HSA parameters are measured, i.e. narrow neck, intertrochanter and femur shaft, respectively (Beck, 2007)*.

## Discussion

In the last decades there has been a great effort toward a more accurate and reliable assessment of the fracture risk in elderly osteoporotic individuals. From this perspective, three-dimensional FE models developed from CT and QCT scans have shown excellent strains and fracture load predictive abilities with respect to *in vitro* experiments (Schileo et al., 2007, 2008a; Grassi et al., 2012). However, CT does not represent the standard imaging technique for osteoporosis screening purposes, and therefore, at present, patient-specific three-dimensional FE models do not represent a clinically attainable risk assessment tool. This is the main reason why there has been growing interests in 2D FE models derived from DXA, which, on the contrary, represents the gold standard imaging technique for osteoporosis diagnosis. Hence, the main purpose of this study was the comparison between the predictive power of 2D and 3D FE models built from DXA and CT images of the same patients. We were interested in investigating how DXA-derived data and analyses, clinically achievable, could integrate the current standard for an enhanced fracture risk estimation.

To accomplish this goal, a risk of fracture index, named $\hat{\text{RF}}$, was analogously computed from the outcomes of both FE analyses. The availability of CT and DXA images for the same set of individuals is not straightforward and, to the authors' knowledge, only (Dall'Ara et al., 2016) dealt with the comparison of DXA and QCT FE models predictive capabilities. Other studies focused on DXA derived FE models (Testi et al., 1999; Langton et al., 2009; Luo et al., 2011, 2018; Op Den Buijs and Dragomir-Daescu, 2011; MacNeil et al., 2012; Naylor et al., 2013; Yang et al., 2014, 2018), although mainly comparing them with experimental tests (Testi et al., 1999; Langton et al., 2009; Op Den Buijs and Dragomir-Daescu, 2011) or comparing DXA and CT in terms of geometric features computation (Cody et al., 2002; Ito et al., 2010; Danielson et al., 2013; Ohnaru et al., 2013). Despite the majority of authors dealing with DXA-based 2D FE models could have access to a BMD local map allowing the straightforward definition of local material properties (Luo et al., 2011, 2018; MacNeil et al., 2012; Naylor et al., 2013; Yang et al., 2014, 2018; Dall'Ara et al., 2016), BMD values restricted to only three ROIs were provided in our case. As a consequence, a procedure postulating a linear relation between image gray values and BMD was proposed in order to obtain a more accurate local material properties assignment, not attainable otherwise. Five different linear relations were firstly defined, aiming to identify the optimal one. The relation defined assuming the minimum BMD value equal to 20% of the average one was considered the most suitable. The so derived $\hat{\text{RF}}$ values were in good agreement with the 3D ones, showing a positive significant correlation (Spearman's ρ = 0.66, *p* < 0.001). Interestingly, also the two HSA variables most relevant to the $\hat{\text{RF}}$ were the same in the 2D and 3D case. This might suggest that geometric features are similarly embedded in DXA and CT-based models. In spite of their intrinsic limitations, the DXA-derived FE models managed indeed to merge and account for both patient-specific geometry and bone mineral properties. This is witnessed by the illustrative shape comparison proposed in Figure 4, where, for instance, patients with comparable T-score but distinct shapes were differently classified by the $\hat{\text{RF}}$. From this perspective, it is the synergic effect of geometry and local material properties which let the 2D $\hat{\text{RF}}$ highlight some osteopenic patients as being at high risk of fracture, while deeming some osteoporotic individuals as not. These results also prospectively emphasize how the T-score predictive ability could be enhanced. T-score, which accounts for the material properties alone, might indeed be supported by the inclusion of the HSA variables highlighted as the most relevant with respect to the $\hat{\text{RF}}$. Without any substantial modifications in the diagnostic procedure, HSA variables, already in hands of clinicians who do not use them, could be thus immediately employed.

Follow-up information unavailability and the limited number of subjects involved represent the main limitations of this study, since they prevented the validation of the implemented models as well as the performance of more robust statistical tests. Of course, the inclusion of the two additional patients did not allow a real validation of the proposed methods, and in spite of the promising outcomes, further investigations would be needed. Although one of the two fractured patients was predicted as at risk of fracture by the 2D analyses, the other was not. However, the explanation could lie in her extremely low BMI, since the applied impact force was reasonably defined as being patient-specific and BMI dependent. Future works might carry out strength calculation on the 2D and 3D models, as in Dall'Ara et al. (2016), even though this would neglect patient-specific data in the impact force definition.

Compared to CT-based three-dimensional models 2D DXA-based FE models certainly have a number of limitations, starting from DXA projective nature, resulting in the overlapping of cortical and trabecular bone on the image plane, going to the approximation of the femur geometry to a plate with constant thickness and boundary conditions applied on the unique image plane. As a consequence, stress and strain distributions may be altered, and the failure starting location might not be fully reliable. Nevertheless, DXA-derived FE analyses have here demonstrated a good agreement with CT-based ones. Besides, they also proved to be able to discriminate patient accounting for the bone mineral properties and patient-specific geometry simultaneously. Being DXA images currently clinically available, the applicability of this method in combination with the T-score is achievable, with the potentiality of integrating and improving the current standards for risk of fracture estimation. Larger cohorts and follow-up data could help in appraising if, combined with T-score, DXA-based analyses show an enhanced ability in discriminating patients at high risk of fracture with respect to T-score alone.

## Data Availability

The datasets generated for this study are available on request to the corresponding author.

## Author Contributions

MT and AA have set up the FE models, have performed numerical simulations, and have worked on parameters optimization. LR contributed to the local BMD map definition and provided all the necessary information regarding DXA imaging technique. GO dealt with the DXA and CT images collection. CB and ALA have supervised the work, giving hints for model validation. CB has specifically addressed biomechanical aspects and has organized the work and its objectives. The paper has been co-written by all the authors.

### Conflict of Interest Statement

The authors declare that this study received funding from TECHNOLOGIC S.r.l. Hologic Italia. The funder contributed to the study by providing all technical details regarding DXA imaging technology and was involved in the local BMD map definition, which partly constituted the design phase of the study. The funder had no role neither in data collection and analysis nor in decision to publish or in manuscript preparation. LR was employed by company TECHNOLOGIC S.r.l. Hologic Italia during the course of the study. The remaining authors declare that the research was conducted in the absence of any commercial or financial relationships that could be construed as a potential conflict of interest.
