# Supplementary material for: Improving the Hip Fracture Risk Prediction Through 2D Finite Element Models From DXA Images: Validation Against 3D Models
Source: Front Bioeng Biotechnol. 2019 Sep 10;7:220. doi: 10.3389/fbioe.2019.00220 (PMC6746936; doi:10.3389/fbioe.2019.00220)
Supplement: Supplementary file 1 [file Table_1.DOCX]

Supplementary Material

**Table S1** Overview of the patient-specific data: age, mass, height, BMI values, Bone Mineral Density (BMD) computed on the whole femur and in the neck region (Neck BMD), maximum and average RF values as well as the 11 non-collinear HSA variables are reported. The HSA variables are presented ordered with respect to their relative relevance to the $\hat{\mathrm{RF}}$, estimated from the cumulative Akaike weights. Neck Shaf Angle (NSA), Buckling Ratio (BR), Width (W), Cross-sectional Moment of Inertia (CSMI), Hip Axis Length (HAL). NN, IT, FS refer to the three locations where HSA parameters are measured, i.e. narrow neck, intertrochanter and femur shaft respectively (Beck, 2007).

| Pat. | Age | Height (cm) | Mass (kg) | BMI | BMD (g/cm^2^) | Neck BMD (g/cm^2^) | $\hat{\mathbf{RF}}$ | Average RF | NSA  (°) | NN BR | NN W  (mm) | NN CSMI  (mm^4^) | HAL  (mm) | FS CSMI  (mm^4^) | IT W  (mm) | | IT BR | IT CSMI  (mm4) | FS W  (mm) | FS BR |
| --- | --- | --- | --- | --- | --- | --- | --- | --- | --- | --- | --- | --- | --- | --- | --- | --- | --- | --- | --- | --- |
| **P1** | **75** | **158** | **55** | **22** | **0.77** | **0.69** | **0.51** | **0.09** | **124.63** | **10.53** | **3.16** | **2.20** | **95.0** | **2.88** | **5.29** | **9.43** | | **9.92** | **2.93** | **3.62** |
| **P2** | **72** | **165** | **82** | **30** | **0.50** | **0.37** | **2.10** | **0.37** | **138.35** | **22.76** | **3.59** | **1.69** | **106.0** | **2.95** | **5.48** | **13.37** | | **7.29** | **3.14** | **5.89** |
| **P3** | **69** | **160** | **55** | **21** | **0.82** | **0.62** | **0.50** | **0.10** | **113.91** | **12.66** | **3.27** | **2.44** | **108.0** | **3.22** | **5.04** | **8.49** | | **8.69** | **2.87** | **2.83** |
| **P4** | **66** | **145** | **66** | **31** | **0.72** | **0.6** | **0.70** | **0.14** | **115.90** | **19.49** | **4.30** | **3.18** | **85.0** | **2.41** | **6.12** | **11.70** | | **9.07** | **2.63** | **2.37** |
| **P5** | **71** | **150** | **58** | **26** | **0.75** | **0.6** | **0.60** | **0.12** | **124.00** | **11.90** | **3.05** | **1.60** | **101.0** | **3.22** | **5.21** | **8.87** | | **12.00** | **3.17** | **4.71** |
| **P6** | **68** | **150** | **41** | **18** | **0.47** | **0.37** | **1.08** | **0.19** | **120.47** | **19.21** | **2.98** | **1.17** | **88.0** | **1.60** | **5.03** | **16.09** | | **4.83** | **2.55** | **4.06** |
| **P7** | **70** | **158** | **53** | **21** | **0.92** | **0.81** | **0.43** | **0.10** | **128.10** | **6.42** | **2.62** | **1.55** | **92.0** | **2.02** | **4.70** | **6.29** | | **8.22** | **2.47** | **1.92** |
| **P8** | **71** | **160** | **71** | **28** | **0.84** | **0.66** | **0.67** | **0.12** | **122.80** | **12.42** | **3.33** | **2.41** | **108.0** | **3.82** | **5.72** | **9.54** | | **14.21** | **3.09** | **3.04** |
| **P9** | **72** | **160** | **56** | **22** | **0.79** | **0.70** | **0.39** | **0.09** | **123.21** | **12.12** | **3.46** | **3.20** | **107.0** | **4.13** | **5.93** | **10.91** | | **13.91** | **3.21** | **3.53** |
| **P10** | **67** | **160** | **62** | **24** | **0.81** | **0.67** | **0.68** | **0.13** | **122.58** | **10.97** | **3.09** | **1.78** | **95.0** | **3.17** | **5.59** | **8.22** | | **13.28** | **2.96** | **3.40** |
| **P11** | **63** | **160** | **85** | **33** | **0.76** | **0.66** | **0.90** | **0.12** | **123.39** | **12.69** | **3.51** | **2.87** | **107.0** | **5.36** | **6.21** | **11.11** | | **18.06** | **3.55** | **4.02** |
| **P12** | **57** | **155** | **63** | **26** | **0.73** | **0.56** | **0.60** | **0.12** | **128.52** | **15.63** | **3.30** | **1.91** | **107.0** | **2.84** | **5.67** | **10.15** | | **12.95** | **2.82** | **3.08** |
| **P13** | **72** | **160** | **73** | **29** | **0.61** | **0.47** | **1.01** | **0.21** | **126.19** | **22.49** | **3.88** | **2.85** | **120.0** | **4.48** | **6.09** | **14.57** | | **13.58** | **3.45** | **5.04** |
| **P14** | **67** | **150** | **74** | **33** | **0.88** | **0.86** | **0.60** | **0.10** | **121.18** | **7.93** | **3.19** | **3.04** | **95.0** | **3.45** | **5.41** | **9.36** | | **10.70** | **2.89** | **2.32** |
| **P15** | **74** | **156** | **51** | **21** | **0.58** | **0.50** | **0.65** | **0.14** | **123.36** | **14.96** | **3.26** | **1.96** | **105.0** | **2.62** | **5.43** | **13.90** | | **8.68** | **3.17** | **5.49** |
| **P16** | **70** | **160** | **75** | **29** | **0.86** | **0.68** | **0.42** | **0.10** | **116.55** | **10.76** | **3.16** | **2.13** | **92.0** | **3.68** | **6.10** | **7.64** | | **21.52** | **2.99** | **2.73** |
| **P17** | **68** | **166** | **69** | **25** | **0.72** | **0.50** | **0.53** | **0.12** | **115.82** | **12.33** | **3.34** | **2.39** | **107.0** | **3.76** | **5.61** | **10.25** | | **14.21** | **3.32** | **4.51** |
| **P18** | **77** | **155** | **61** | **25** | **0.67** | **0.54** | **0.69** | **0.12** | **123.76** | **15.84** | **3.41** | **2.09** | **109.0** | **4.26** | **6.31** | **12.51** | | **15.43** | **3.24** | **3.95** |
| **P19** | **55** | **162** | **91** | **35** | **0.94** | **0.81** | **0.54** | **0.11** | **129.94** | **10.23** | **3.40** | **3.11** | **108.0** | **4.70** | **5.88** | **8.32** | | **18.01** | **3.18** | **2.65** |
| **P20** | **74** | **157** | **62** | **25** | **0.83** | **0.71** | **0.49** | **0.09** | **122.68** | **11.27** | **3.21** | **2.39** | **97.0** | **4.27** | **5.36** | **9.09** | | **12.76** | **3.27** | **3.98** |
| **P21** | **65** | **145** | **53** | **25** | **0.77** | **0.58** | **1.13** | **0.14** | **132.62** | **10.61** | **2.86** | **1.42** | **91.0** | **1.43** | **4.96** | **7.41** | | **7.46** | **2.16** | **1.46** |
| **P22** | **72** | **167** | **63** | **23** | **0.68** | **0.52** | **0.76** | **0.14** | **124.14** | **16.17** | **3.29** | **1.89** | **108.0** | **3.35** | **5.98** | **10.89** | | **14.66** | **3.06** | **4.11** |
| **P23** | **79** | **152** | **45** | **19** | **0.69** | **0.56** | **0.67** | **0.11** | **124.17** | **13.57** | **3.21** | **1.92** | **102.0** | **3.18** | **5.25** | **11.61** | | **9.46** | **3.10** | **3.75** |
| **P24** | **61** | **150** | **72** | **32** | **0.80** | **0.66** | **0.63** | **0.12** | **126.70** | **12.73** | **3.32** | **2.34** | **99.0** | **2.97** | **5.67** | **8.42** | | **13.80** | **2.92** | **3.25** |
| **P25** | **72** | **152** | **55** | **24** | **0.61** | **0.52** | **0.96** | **0.13** | **124.37** | **14.01** | **3.08** | **1.49** | **103.0** | **2.45** | **5.42** | **12.59** | | **9.47** | **3.08** | **5.69** |
| **P26** | **81** | **155** | **55** | **23** | **0.60** | **0.54** | **0.90** | **0.15** | **127.49** | **14.24** | **3.31** | **1.92** | **107.0** | **2.89** | **5.93** | **13.19** | | **10.31** | **3.04** | **4.86** |
| **P27** | **71** | **163** | **79** | **30** | **0.99** | **0.77** | **0.48** | **0.09** | **129.47** | **11.52** | **3.82** | **4.69** | **102.0** | **4.28** | **6.01** | **8.05** | | **17.90** | **3.02** | **2.10** |
| **P28** | **79** | **143** | **46** | **22** | **0.55** | **0.45** | **0.92** | **0.16** | **127.48** | **17.21** | **3.35** | **1.70** | **96.0** | **2.04** | **5.29** | **13.54** | | **7.79** | **2.84** | **4.49** |
| **P29** | **71** | **135** | **70** | **38** | **0.69** | **0.59** | **0.87** | **0.12** | **117.00** | **17.70** | **3.62** | **2.44** | **87.0** | **3.14** | **6.34** | **9.70** | | **4.51** | **2.85** | **2.80** |
| **P30** | **75** | **168** | **45** | **16** | **0.58** | **0.63** | **0.59** | **0.12** | **123.00** | **19.90** | **3.95** | **2.28** | **103.0** | **2.26** | **6.00** | **16.30** | | **3.32** | **2.99** | **5.37** |

**Local BMD mapping procedure**

Due to the lack of a pixel by pixel Bone Mineral Density (BMD) map, a linear relation between the grey value (GV) and the BMD of each pixel was postulated in order to extract, although approximately, the missing local BMD map for inhomogeneous material properties assignment. Only the 3 BMD values output by the DXA software for clinical purposes were available, i.e. the mean BMD values at the neck, trochanteric and intertrochanteric Region Of Interest (ROI) (Fig. S1). Hence, the linear relation assessment was carried out for each ROI individually. In order to define it, at least two points were needed. The first one was determined considering the available ROI-specific mean BMD value, which was matched to the average GV value computed in the corresponding ROI neglecting zero GVs. The second point was defined estimating the minimum BMD value as a percentage of the mean value, and establishing its correspondence with the minimum GV value, always equal to 0 (Fig. S2).


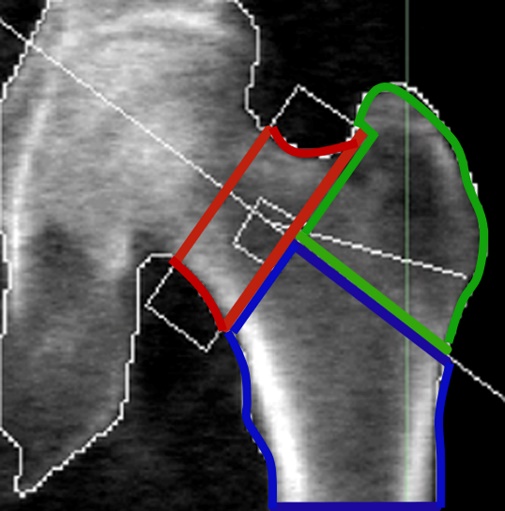


Figure S1 A typical proximal femur DXA image with highlighted the Regions Of Interest (ROI) accounted for within the DXA software: the neck (red), trochanter (green), intertrochanter (blue).


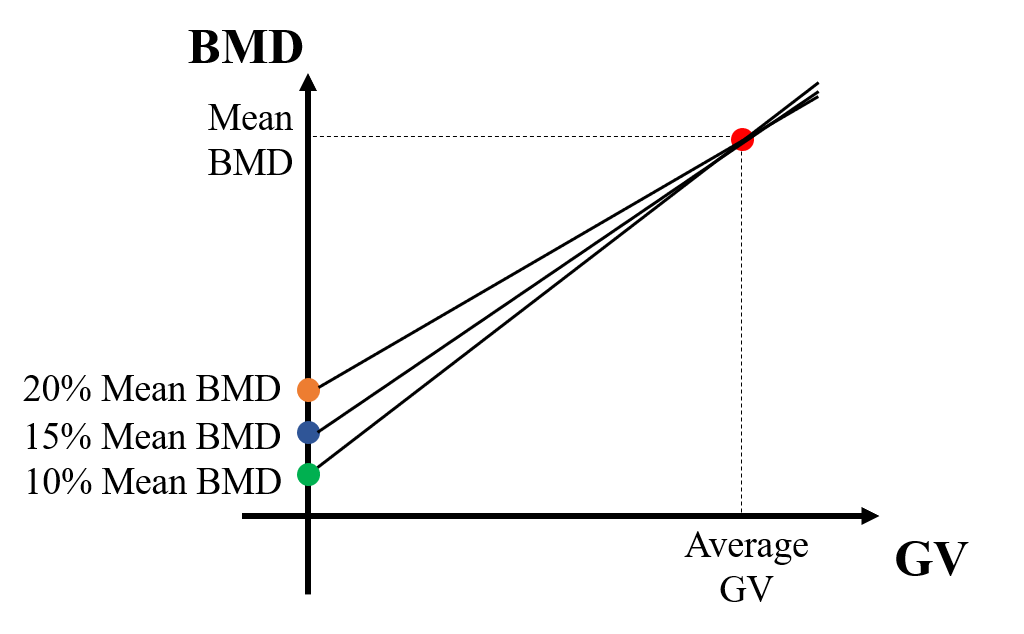


Figure S2 An explanatory graph illustrating how the linear relation between pixel GV and BMD was defined at each ROI. The line was defined with respect to two points: one establishing the correspondence between the provided mean BMD value per each ROI with the average GV computed accounting for the non-zero GV at each ROI (red dot), the other one estimating the minimum BMD value as a function of the mean one (orange, blue, green dots), and corresponding to the respective minimum GV (which was always equal to 0).

Fig. S2 provides a schematic representation of the adopted methodology. In addition to the point defined on the basis of the given mean BMD value, just three different minimum BMD values are shown for ease of visualization. The unknown minimum BMD value, equivalent to the line intercept, was actually determined in five different trials as 10%, 15%, 20%, 25% and 30% of the average BMD. Having two points set, the intercept and the slope of the line were defined, leading to the full definition of the linear relation between BMD and GV. This procedure was carried out at each different ROI (neck, trochanter and intertrochanter) for each patient. In order to extract one unique patient-specific linear relation, the ROI-specific intercept and slope values were eventually averaged.


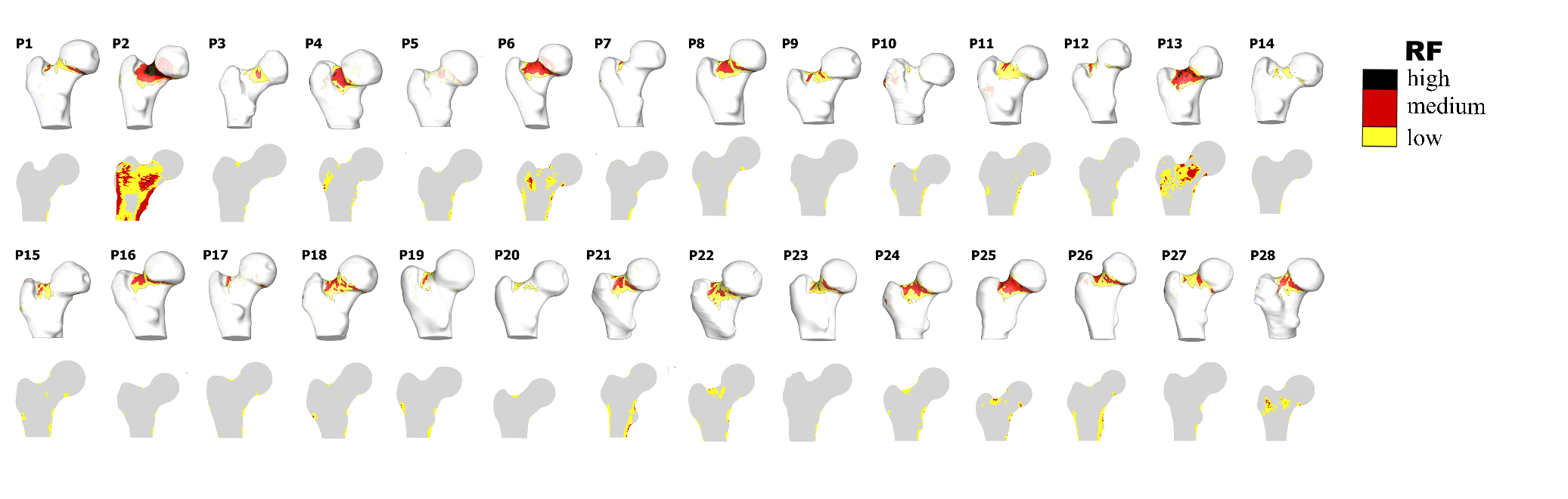


Figure S3 Comparison of the RF contour plots for the CT-based (1^st^ and 3^rd^ row) and DXA-based (2^nd^ and 4^th^ row) models.
